# Using latent class analysis to identify clinical features of patients with occlusive myocardial infarction: Preangiogram prediction remains difficult

**DOI:** 10.1002/clc.23755

**Published:** 2022-02-08

**Authors:** Charles Knoery, Katie. A. McEwan, Matthew Manktelow, Jonathan Watt, Jamie Smith, Aleeha Iftikhar, Khaled Rjoob, Raymond Bond, Victoria McGilligan, Aaron Peace, Anne McShane, Janet Heaton, Stephen J. Leslie

**Affiliations:** ^1^ Division of Rural Health and Wellbeing, Institute of Health Research and Innovation, Centre for Health Science University of the Highlands and Islands Inverness UK; ^2^ Cardiac Unit, Raigmore Hospital NHS Highland Inverness UK; ^3^ Centre for Personalised Medicine, Biomedical Sciences Research Institute Ulster University Londonderry Northern Ireland UK; ^4^ School of Computing, Jordanstown Campus Ulster University Newtownabbey Northern Ireland UK; ^5^ Cardiology Department Altnagelvin Hospital Londonderry Northern Ireland UK; ^6^ Emergency Department Letterkenny University Hospital Donegal Ireland

**Keywords:** coronary artery, myocardial infarction, occlusion/occlusive

## Abstract

**Background:**

Treatment decisions in myocardial infarction (MI) are currently stratified by ST elevation (ST‐elevation myocardial infarction [STEMI]) or lack of ST elevation (non‐ST elevation myocardial infarction [NSTEMI]) on the electrocardiogram. This arose from the assumption that ST elevation indicated acute coronary artery occlusion (OMI). However, one‐quarter of all NSTEMI cases are an OMI, and have a higher mortality. The purpose of this study was to identify features that could help identify OMI.

**Methods:**

Prospectively collected data from patients undergoing percutaneous coronary intervention (PCI) was analyzed. Data included presentation characteristics, comorbidities, treatments, and outcomes. Latent class analysis was undertaken, to determine patterns of presentation and history associated with OMI.

**Results:**

A total of 1412 patients underwent PCI for acute MI, and 263 were diagnosed as OMI. Compared to nonocclusive MI, OMI patients are more likely to have fewer comorbidities but no difference in cerebrovascular disease and increased acute mortality (4.2% vs. 1.1%; *p* < .001). Of OMI, 29.5% had delays to their treatment such as immediate reperfusion therapy. With latent class analysis, while clusters of similar patients are observed in the data set, the data available did not usefully identify patients with OMI compared to non‐OMI.

**Conclusion:**

Features between OMI and STEMI are broadly very similar. However, there was no difference in age and risk of cerebrovascular disease in the OMI/non‐OMI group. There are no reliable characteristics therefore for identifying OMI versus non‐OMI. Delays to treatment also suggest that OMI patients are still missing out on optimal treatment. An alternative strategy is required to improve the identification of OMI patients.

## 1 INTRODUCTION

Despite advances in diagnosis and treatment, acute coronary syndromes (ACS) remain a leading cause of mortality. In Scotland, ACS is the cause of 6600 deaths making it the leading cause of death.^1^ The most severe form of ACS is when a coronary artery is occluded, commonly presenting as a ST‐elevation myocardial infarction (STEMI) with a high short‐term mortality (9.7% of all hospital patients). Interestingly, non‐ST elevation myocardial infarction (NSTEMI), which during the acute phase are less fatal but have a higher 1‐year mortality (18.7% vs. 8.4% of hospital survivors).^2^ The traditional explanation of differentiation and mortality rates between STEMI and NSTEMI, was that ST elevation on the electrocardiogram (ECG) represents acute coronary artery occlusion with a large area of cardiac myocardium with no blood flow and therefore an increased short‐term mortality.^3^ In contrast, NSTEMI patients tend to be older with multivessel disease and increased premyocardial infarction (MI) comorbidities but only have partial coronary artery occlusion, giving a potential explanation to why they have increased long‐term mortality but lower short‐term mortality than STEMI.^4^

However, there is increasing evidence that there is a subset of NSTEMI patients who do have acute coronary artery occlusion.^5^, ^6^, ^7^, ^8^ Meta‐analysis of studies looking at angiographic data of NSTEMI patients found that 25.5%–39% of NSTEMI patients have coronary artery occlusion and this is associated with increased short and long‐term mortality.^5^, ^9^ There is also an increase in mortality in comparison to STEMI patients, as due to the lack of ST‐elevation on the ECG, these patients may be mis‐triaged and do not receive timely reperfusion therapy such as percutaneous coronary intervention (PCI) or thrombolysis.^10^ There is no clear way to clinically distinguish between occlusive MI and nonocclusive MI before angiography as ST‐elevation appears nonspecific for coronary artery occlusion and troponin is raised in any cause of myocardial necrosis or turnover regardless of coronary artery occlusion.^11^ Clearly, there is increased imperative to classify ACS as occlusive MI and nonocclusive MI. In turn, there is an obvious need to identify features that distinguish between occlusive MI and nonocclusive MI.^12^

The aim of this study was to use a form of unsupervised learning called latent class analysis to analyze the demographics of patients presenting with ACS to identify if there were, differing features in patients with occlusive and nonocclusive MI that may in turn, improve preangiogram triage.

## 2 METHODOLOGY

### 2.1 Setting

This was a single‐center, retrospective case‐control study in a PCI unit based at a rural regional center in a hospital in the North of Scotland. The hospital covers a large geographical area (32 500 km^2^) with a dispersed population of approximately 250 000 and provides a tertiary cardiology service to several secondary district hospitals. PCI data was collected as part of the British Cardiovascular Intervention Society (BCIS) continuous national audit which includes over 100 data points from all patients undergoing PCI including patient demographics, PCI justification, complications, and outcomes. Due to the large geographical area and a PCI lab that is open on working hours (Monday–Friday, 9 a.m.–5 p.m.), any STEMI patients who are greater than 2 h traveling distance to the PCI center or present out with working hours, are thrombolysed.

### 2.2 Study design and data set collection

In this study, data from 2015 to 2019 were analyzed after the removal of identifiable data such as names, address, dates of procedure, and date of birth.

The data were split into several predefined categories. Categories included numbers of occlusive MI/nonocclusive MI and occlusive MI without ST elevation on ECG (occlusive NSTEMI) and occlusive MI with ST‐elevation on ECG (occlusive STEMI). The differentiation between NSTEMI and STEMI was ST elevation on ECG fulfilling the European Society of Cardiology universal definitions.^11^ Presentation demographics of, age, gender, ST elevation, cardiogenic shock, and out‐of‐hospital cardiac arrest were recorded. Several categories were refined by combining various subgroups. For instance, “ex‐smoker” and “current smoker” were combined into one category. Comorbidities included prior MI, prior coronary artery bypass graft, diabetes, peripheral vascular disease, hypercholesteremia, hypertension, cerebrovascular disease, vascular heart disease, and renal disease. Risk factors included smoking and family history of coronary artery disease. Treatments recorded included urgent PCI (with 72 h of symptom onset), primary PCI (within 12 h of symptom onset), rescue PCI (following failed thrombolysis), thrombolysis, aspirin treatment, P2Y12 inhibitor treatment. A separate category was created for immediate reperfusion therapy that included all patients that had thrombolysis and/or primary PCI. Outcomes recorded included procedure complications, episode mortality, left ventricular ejection fraction (further split into over 50%, between 30% and 50% and less than 30%).

To determine whether a patient had acute coronary artery occlusion, the recorded stenosis status of the coronary artery before and after PCI was compared. In the data set, the stenosis pre‐ and post‐PCI of the coronary arteries left main stem, left anterior descending artery (LAD—proximal and distal), right coronary artery (RCA), and left circumflex (LCx) were recorded. Acute coronary artery occlusion was identified on angiogram if the pre‐PCI stenosis was 100% and post‐PCI stenosis was 0%–49%.

### 2.3 Statistical analysis

The data were entered onto SPSS™ version 25 (IBM) for statistical analysis. For initial descriptive and inferential statistical analysis, crosstabs with Pearson *χ* ^2^ testing were used to determine for categorical variables. Binary logistical regression was used if the independent variable had more than two levels. Fisher's exact test was used when there were categories that had values less than five and an independent sample *t*‐test was used to determine statistical significance for continuous variables. A *p*‐value less than .05 was considered significant.

Latent class analysis was performed, using MPlus version 8.6 (Muthén & Muthén, to determine whether distinct patterns of presentation and history were associated with acutely occlusive MI. Latent class analysis is an unsupervised learning statistical framework for model‐based clustering and identifying subgroups or typologies that characterize heterogeneity in a population. It segments a data set into classes based on case similarities for a particular set of variables or for dichotomous data, “indicators”; cases are assigned a probability of class membership based on maximum likelihood estimation. Each class, therefore, has associated with it a set of probabilities describing the likelihood of a member for values of the indicator variables, which together describe the characteristics of the class.

Given the relatively large number of indicator variables compared to cases, the data set was rendered more tractable for analysis by dichotomizing certain variables (LVEF and New York Heart Association symptoms). An initial assessment of the contribution of the various indicator variables to the model also identified smoking status as being of low significance in partitioning the classes, and it was therefore removed.

Class membership is considered a “latent” or unobserved variable, that may capture underlying phenotypes not accessible through more traditional analysis.^13^ Overall goodness of fit of a particular number of classes to the data set is assessed by various measures and statistical tests, and robust confidence intervals (CIs) for indicators can be calculated. Further details of the analysis conducted are provided in the Supporting Information. As an omnibus test, the Wald test does not identify between which classes the significant difference arises; therefore, *z* tests with a Holm–Bonferroni correction were used to establish where significant differences occur.

### 2.4 Ethics

Ethical permission for the research was obtained from the NHS Highland Caldicott committee. As the data had already been collected for audit purposes and this study did not involve any patients contact or intervention, full ethical permission was not required.

## 3 RESULTS

A total of 1412 underwent PCI for acute MI, and 263 had occlusive MI on angiogram (Table 1). Of these, 510 (36.1%) patients were classified as a STEMI compared to 902 (63.9%) who were classified as NSTEMI. There were 263 (18.6%) patients with an occlusive MI and 1149 (81.4%) patients with nonocclusive MI. Table 2 lists the demographics and outcomes of the occlusive MI and nonocclusive MI cohort as well as the outcomes of the NSTEMI occlusive MI and STEMI occlusive MI cohorts.

**Table 1:** Separation into four groups of NSTEMI/STEMI compared to occlusive myocardial infarction/nonocclusive myocardial infarction

	NSTEMI (%)	STEMI (%)	Total
Nonocclusive myocardial infarction	*N* = 806 (89.4%)	*N* = 343 (67.3%)	1149
Occlusive myocardial infarction	*N* = 96 (10.6%)	*N* = 167 (32.7%)	263
Total	902 (100%)	510 (100%)	1412

Abbreviations: NSTEMI, non‐ST elevation myocardial infarction; STEMI, ST‐elevation myocardial infarction.

**Table 2:** Features of occlusive MI versus nonocclusive MI

	Total		OMI		Non‐OMI		OMI NSTEMI			OMI STEMI		
	No.	%	No.	%	No.	%	*p*‐Value	No.	%	No.	%	*p*‐Value
Number	1412	100	263	18.6	1149	81.4		96	36.5	167	63.5	
Age (mean)	66	–	65.5	–	66.7	–	.122	64.2	–	66.3	–	.199
Gender (male)	1023	72.5	191	72.6	832	72.4	.944	70	72.9	121	72.5	.936
ST elevation			167	63.5	343	29.9	**<.001**	–	–	–	–	
Cardiogenic shock	48	3.4	24	9.1	24	2.1	**<.001**	3	3.1	21	12.6	**.01**
OOH cardiac arrest	40	2.8	14	5.3	26	2.3	**.007**	3	3.1	11	6.6	.229
Comorbidities												
Prior MI	357	25.3	53	20.2	304	26.5	**.034**	21	21.9	32	19.2	.597
Prior CABG	92	6.5	10	3.8	82	7.1	**.048**	6	6.3	4	2.4	.116
Diabetes	298	21.1	39	14.8	259	22.5	**.006**	11	11.5	28	16.8	.244
Peripheral vascular disease	14	1	9	3.4	52	4.5	.427	4	4.2	5	3	.615
Hypercholesteraemia	98	6.9	11	4.2	87	7.6	.051	8	8.3	3	1.8	**.020** a
Hypertension	726	51.4	120	45.6	606	52.7	**.037**	45	46.9	75	44.9	.758
Cerebrovascular disease	134	9.5	27	10.3	107	9.3	.634	9	9.4	18	10.8	.718
Valvular heart disease	14	1	1	0.4	13	1.1	.216^b^	0	0	1	1.6	.635
Renal disease	63^a^	4.5	7	2.7	56	4.9	.124	4	4.2	3	1.8	.222
Risk factors												
Family history of CAD	512^c^	36.5	95	36.1	417	36.5	.994	43	44.8	52	31.7	0.034
Smoker	921^d^	65.1	170	64.6	751	65.4	.931	67	69.8	103	61.7	0.271
Treatment												
Urgent PCI	347	24.6	78	29.7	986	85.8	**<.001**	65	67.7	13	7.8	**<.001**
Emergency PCI	1064	75.4	185	70.3	162	14.1	**<.001**	31	32.3	154	92.2	**<.001**
Immediate reperfusion therapy	487	34.5	188	71.5	299	26	**<.001**	31	32.3	157	94	**<.001**
Prior thrombolysis	190	13.5	18	6.8	172	15	**<.001**	3	3.1	15	9	.07
Outcomes												
Procedure complication	78	5.5	19	7.2	59	5.1	0.181	8	8.3	11	6.6	.598
Episode mortality	24	1.7	11	4.2	13	1.1	**<.001**	1	1	10	6	.061
LVEF > 50%	263	18.6	80	30.4	637	55.4	**<.001**	37	38.5	43	25.7	.503
LVEF: 30%–50%	717	50.8	147	55.9	423	36.8	**<.001**	52	54.2	95	56.9	**<.001**
LVEF < 30%	570	40.4	31	11.8	71	6.2	**<.001**	5	5.2	26	15.7	**.001**

Abbreviations: CABG, coronary artery bypass graft; CAD, coronary artery disease; LVEF, left ventricular ejection fraction; MI, myocardial infarction; OOH, out of hours; OMI, occlusive myocardial infarction; PCI, percutaneous coronary intervention.

^a^ 3 Cases missing info.

^b^ Fisher test as 1 count less than 5.

^c^ 10 Cases missing info.

^d^ 3 Cases missing info.

**Figure 1:**
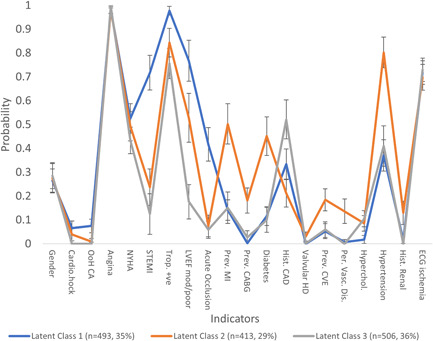
Latent class analysis when split into three classes based on maximum likelihood estimation

In the initial approach, a latent class analysis model was derived for all the indicators together, determining that a model with three classes fit the data best (Figure 1). Thus, for example, a member of latent class 2 has an 80% chance of having a history of hypertension, but only a 24% chance of having a STEMI. Latent class 2 is characterized as an older, comorbid cluster, while latent class 1 has significantly higher rates of STEMI and acute occlusion.

Considering those NSTEMI patients found to be acutely occluded, the majority were assigned to Class 1, along with the STEMI patients. For Class 1, *n* = 59; for Class 2, *n* = 13; and for Class 3, *n* = 26. The associated odds ratio was 7.663 for latent class 1 compared to latent class 2 (*p* = .0316, 95% CI: 3.075–19.096) and 6.335 for latent class 1 compared to latent class 3 (*p* = .0223, 95% CI: 2.693–14.996). The odds ratio of 0.829 between latent classes 2 and 3 was not significant (*p* = .1011, 95% CI: 0.251–2.740).

The above results indicate commonality of history and presentation for many patients experiencing acute occlusion, but do not signpost towards identification of acutely occluded NSTEMI. We, therefore, forced separation into groups according to ST elevation and occlusion status. Consideration of model goodness of fit indicates that the optimal solution was obtained by deriving two classes per group.

**Figure 2:**
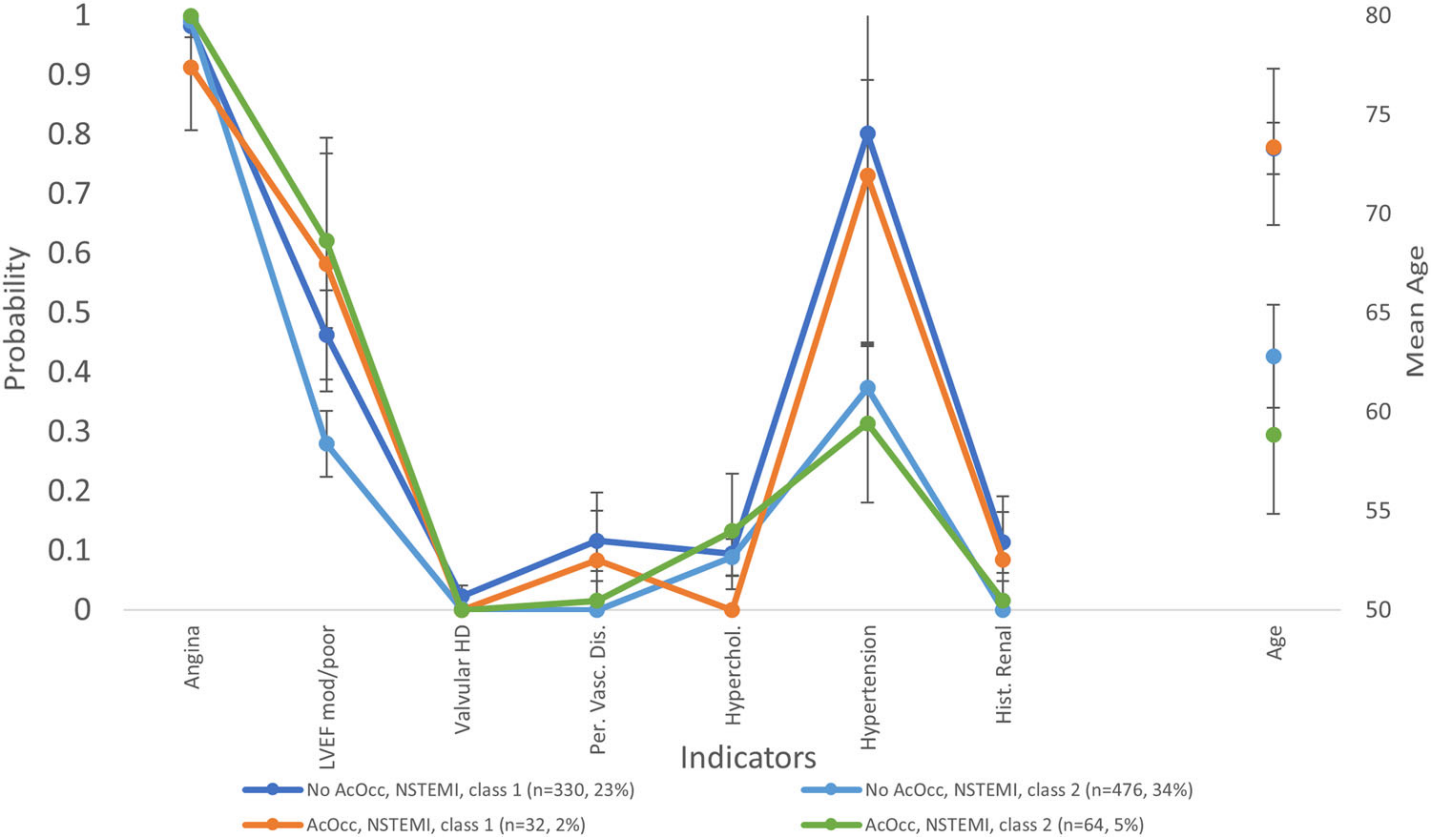
Latent class indicator probabilities differing between classes for the two NSTEMI groups

A Wald test indicated that for the majority (*n* = 16) of the 19 variables, there was a significant difference between classes. As our particular focus was on distinguishing acute occlusion in NSTEMI, Figure 2 illustrates only those indicators found to differ significantly between classes in the NSTEMI groups.

The significant differences with NSTEMI arose between different classes with the same occlusion status, rather than indicator probabilities being usefully associated with occlusion status. This indicated that the use of latent class analysis was not able to identify features that differentiated between occlusive and nonocclusive MI.

We then performed a sensitivity analysis using logistical regression with same variables used in the latent class analysis for acute occlusion in patients with NSTEMI. In this model with a cut‐off value of 0.3 and in NSTEMI patients, the sensitivity for acute occlusion was 97.8% and specificity was 11.8%. The model explained 11.4% (Nagelkerke *R* ^2^) of the variance in acute occlusion, correctly identifying 88.6% of cases. However, the only significant factors associated with acute occlusion and absence of diabetes (odds ratio [OR]: 0.43, 95% CI: 0.21–0.89, *p* = .022) and moderate LVEF (OR: 3.03, 95% CI: 1.87–4.91, *p* < .001).

## 4 DISCUSSION

This retrospective analysis of 4 years of angiographic data at a single‐center cardiac catheterization laboratory found a significant increase in hemodynamic instability, fewer comorbidities, and increased short‐term mortality and morbidity in occlusive MI patients, indicating subgroup at who are at increased risk of unfavorable outcomes. Out of 263 patients with occlusive MI, 29.5% had delays to their treatment (i.e., not having emergency PCI or thrombolysis), which is similar to previous findings that one‐quarter of all NSTEMI have occlusive MI and are misclassified.^5^ This finding suggests that occlusive MI patients are still missing out on optimal treatment.

When comparing occlusive MI against nonocclusive MI, occlusive MI features often were concordant with STEMI. Occlusive MI patients were significantly more likely to have similar complications to STEMI such as cardiac arrest and reduced LVEF. They were also more likely to have an initial detectable troponin compared to nonocclusive, perhaps representing the increased size of the infarct. However, patients with nonocclusive MI were more likely to receive thrombolysis (15% vs. 6.8%). This finding would make sense as thrombolysis would increase the likelihood of reperfusion of an occluded artery and for the vessel to appear nonocclusive when the patient undergoes PCI and thus initial appearances may have been of occlusive MI. Occlusive MI patients were also significantly less likely to have comorbidities. A lower prevalence of diabetes and hypertension has already been described in occlusive MI but unlike our results they also found occlusive MI patients younger and smokers.^14^ Conversely, diabetes and hypertension have been found to be independent risk factors for occlusion as part of the CHA2DS2‐VASc scoring system along with previous stroke and vascular disease.^15^ Although numbers in the study were low, further research is required to clarify the risk factors for occlusion.

As around one‐quarter of all NSTEMI have acute occlusion with associated increased mortality, we would expect the same in our data.^5^ In the cohort analyzed in this study, 36.5% (*n* = 96) of OMI were classified as NSTEMI according to ECG and only 32.4% (*n* = 31) received immediate reperfusion therapy compared to 94.0% (*n* = 157) of occlusive STEMI (*p* < .001). Apart from a higher rate of hypercholesteremia (8.3% vs. 1.8%; *p* = .02), these patients did not have any differing characteristics compared to occlusive STEMI. There was also no significant difference in mortality and the occlusive STEMI patients were significantly more likely to have a reduced LVEF. This would be expected as occlusive NSTEMI is more associated with LCx and RCA occlusion and thus less likely to cause LV dysfunction.^5^, ^16^

When comparing similarities and differences between occlusive MI and known STEMI patient cohort, they were broadly similar with a higher percentage likely to be unstable, less comorbidities, and higher risk of death and morbidity.^2^ A potential difference was age, with occlusive MI patients showing no statistical difference in age unlike the STEMI cohorts who are often younger than the NSTEMI cohort.^2^ Therefore, suspicion of occlusive MI should not be influenced by the patient's age. Occlusive MI patients also had no statistical difference in cerebrovascular disease compared to nonocclusive MI patients unlike STEMI and NSTEMI, again indicating a possibility that occlusive MI patients include a frailer patient cohort.

Latent class analysis provided no clear differences between the occlusive MI and nonocclusive MI groups. While some significant differences were detected in the history and presentation of these patients when clustered by latent class analysis, these differences largely correspond to classes, rather than distinguishing between occlusive and nonocclusive MI. This detailed unsupervised learning latent class analysis, provides further evidence that there are no clear distinguishing current features in indicators that can reliably differentiate between patients with occlusive and nonocclusive MI. The lack of reliable indicators suggests that identification of occlusive MI will be overly reliant on additional diagnostic technology such as ECG and biomarkers.

### 4.1 Limitations

This study had several limitations. Importantly, it is data from a single center and under the influence of regional and local population variations. Although the population of the Scottish Highlands has a unique mixture of extremely remote communities and several large towns providing a mixed demographic, comparison to other communities must be taken with caution.

By definition, occlusive MI requires coronary artery occlusion, which is only visible on angiography. Yet coronary artery occlusion is a transient event with an estimated 22% of coronary artery occlusion self‐dissipating between 4 and 12 h from onset, either presumably through the resolution of vasospasm or dissolution of the thrombosis.^17^ So, it is conceivable that may have occlusive MI initially, may be reclassified as nonocclusive MI by the time they undergo PCI and that the numbers of patients with occlusive MI in this study may be underrepresented. It is still important to identify occlusive MI with dynamic changes as there is no guarantee that their coronary artery occlusion will resolve spontaneously as it may reoccur.^18^ Therefore it is vital that all patients with occlusive MI are considered urgent reperfusion therapy such as PCI or thrombolysis.^19^

Additionally, the hospital where the data were analyzed has a cardiac catherization laboratory open only during working hours Monday–Friday, which will likely provide difference results in comparison to a 24/7 PCI center. In addition, due to operational working hours and large remote community that the hospital covers, thrombolysis is still readily used if access of a cardiac catherization lab is greater than 120 min away.

Finally, the data is limited to the data collected at the BCIS audit. There is no long‐term data on survival or morbidity such as 30 day or 1‐year mortality. In addition, the data lacks data on specific symptoms or clinical signs that may help guide diagnosis. However, the similarities in presentation between occlusive MI in cardiogenic shock and prior OOH cardiac arrest would suggest that symptoms and clinical features are similar due to the same pathophysiological process in occlusive MI and the majority of STEMI patients.^20^

## 5 CONCLUSION

Overall although the occlusive MI cohort are broadly similar to known STEMI patient characteristics there are some subtle differences such as an older and frailer patient cohort in the occlusive MI group. This could be an additional factor in explaining why occlusive MI patients have a higher mortality as well as larger full‐thickness infarcts from the increased cardiac myocyte necrosis from total coronary occlusion.^21^

With increased knowledge of the features of occlusive MI, improved and faster diagnosis such as computer ECG analysis and improved education could potentially improve mortality by triaging occlusive MI patients directly for reperfusion therapy such as emergency PCI or thrombolysis. Reclassification of the ECG findings for occlusive MI can lead to increased occlusive MI identification and improved mortality.^22^ Demographic features of occlusive MI, along with ECG analysis and potentially biomarkers could combine into a clinical decision support system to help guide clinicians to identify occlusive MI.^23^ However, as revealed by latent class analysis, there is no reliable distinguishing features between occlusive and non‐occlusive MI. Before novel innovations are available, serial ECGs provide a potential insight into the dynamic nature of coronary artery occlusion and the need for emergency reperfusion therapy if showing indicative changes such as evolving ST elevation.^4^, ^24^

More research is needed to classify the diagnostic features of occlusive MI to help distinguish from noncritical or nonocclusion of the coronary artery. This study adds to the current consensus that although occlusive MI is most critical form of ACS, we are without a robust form of identification.

## CONFLICT OF INTERESTS

The authors declare that there are no conflict of interests.

## Supporting information

Supporting information.

## Data Availability

The data that support the findings of this study are available from the corresponding author upon reasonable request.
