# Supplementary material for: Using latent class analysis to identify clinical features of patients with occlusive myocardial infarction: Preangiogram prediction remains difficult
Source: Clin Cardiol. 2022 Feb 8;45(2):231–8. doi: 10.1002/clc.23755 (PMC8860484; doi:10.1002/clc.23755)
Supplement: Supplementary file 1 — Supporting information. [file CLC-45-231-s001.docx]

# Supplemental information

## Initial analysis (3 class model)

Data preparation: the LVEF and NYHA variables were dichotomized according to (LVEF>=50% = 0; LVEF<50% = 1) and NYHA (NYHA classifications 1&2 = 0; NYHA classifications 3&4 = 1). Initial exploratory analysis using the “entropy” option in MPlus determined that the contribution of the “smoking” indicator to the model was somewhat less than that of the other variables; since reducing the number of variables made analysis more tractable, it was therefore excluded.

Determination of the number of classes generated the following results:

Table 1:Generated model fit parameters for various numbers of classes, unrestricted LCA

| Model  (Number of latent classes) | Loglikelihood | Best Log replicated  (Yes/No) | Num. free parameters | AIC | BIC | SSABIC | LMR-LRT (*p*) | Entropy | BLRT (*p*) |
| --- | --- | --- | --- | --- | --- | --- | --- | --- | --- |
| 1 | -16864.593 |  |  |  |  |  |  |  |  |
| 2 | -16600.692 | yes | 44 | 33289.384 | 33520.505 | 33380.733 | 0.0000 | 0.550 | 0.0000 |
| 3 | -16415.411 | yes | 66 | 32962.822 | 33309.504 | 33099.846 | 0.0000 | 0.588 | 0.0000 |
| 4 | -16352.388 | yes | 88 | 32880.676 | 33342.919 | 33063.375 | 0.0844 | 0.588 | 0.0000 |

The above results are ambiguous between 3 and 4 classes. Nylund, Asparouhov and Múthen propose that the Bootstrap Likelihood Ratio test (BLRT; implemented in MPlus as “Tech14”) is more reliable than the adjusted Lo-Mendell-Rubin Likelihood Ratio test^1^.They further propose the Bayesian Information Criterion (BIC) as the most informative information criteria in determining the number of classes, but consider it somewhat less reliable than the BLRT overall. For this initial analysis we wished to be parsimonious in the number of classes, and chose 3 classes as indicated by BIC and LMR-LRT. Robust confidence intervals are generated using the “cinterval” command.

To assess the odds ratios of acutely occluded NSTEMI with regard to the determined classes, a new indicator variable (“nstemiocc”) was defined, set to 1 for cases of acutely occluded NSTEMI and 0 otherwise. The model was then rerun with thresholds for the other indicators fixed to values determined in the initial analysis, and confidence intervals extracted and p-values derived from the output.

Cross-validation in Latent Class models is somewhat more complex than in other forms of unsupervised learning, due to the statistical model framework underpinning the various measures of goodness of fit used. For this purpose, we used a method using multigroup analysis informed by Collins & Lanza^2^. The dataset was randomly split into two groups with a filter variable using SPSS’s “Select Cases” option, which were then assessed for “measurement invariance” – that the two populations possess the same latent class structure. This was assessed by determining two nested models, using the “knownclass” option in MPlus to define two groups in each model, with each group having three classes. In one model, the parameters for each group’s classes were free to differ (the “unrestricted” model), while in the other (the “restricted” model) they were constrained to be equal across groups. The model fit was then assessed, as shown in table 2 below:

Table 2: Measurement invariance for the 3 class model

| Model | Loglikelihood | Num. free parameters | AIC | BIC | SSABIC |
| --- | --- | --- | --- | --- | --- |
| 2 group, 3 class – Unrestricted | -17363.496 | 130 | 34986.993 | 35669.852 | 35256.889 |
| 2 group, 3 class - Restricted | -17393.449 | 67 | 34920.898 | 35272.833 | 35059.998 |

Measurement invariance is usually established statistically by means of a difference χ^2^ test. However, owing to the large number of indicators in the model, the χ^2^ test cannot be computed because the frequency table for the latent class indicator model part is too large. A comparison of the information criteria indicates that the restricted model (where the same parameters apply across both groups) is a more optimal balance of model fit and parsimony. An examination of the three class solution in table 1 indicates it is likewise superior. This, together with the very similar parameters determined by all three models, indicate the three class solution is likely optimal.

## Analysis by MI group

The “knownclass” option was used to define four fixed classes according to a new variable, “MIgroup”. The number of classes each known class should be divided into was determined according to the fit parameters below:

Table 3: Generated fit parameters for models with 4 known groups, unrestricted LCA

| Model  (Number of latent classes) | Loglikelihood | Best Log replicated  (Yes/No) | Num. free parameters | AIC | BIC | SSABIC | LMR-LRT (*p*) | Entropy | BLRT (*p*) |
| --- | --- | --- | --- | --- | --- | --- | --- | --- | --- |
| 1 | -16635.186 | yes | 80 | 33430.371 | 33850.592 | 33596.461 | n/a | 1.000 | n/a |
| 2 | -16344.188 | yes | 157 | 33002.375 | 33827.059 | 33328.327 | unavailable | 0.586 | unavailable |
| 3 | -16231.895 | no | 231 | 32931.789 | 34160.936 | 33417.602 | unavailable | 0.624 | unavailable |

With likelihood ratio tests unavailable for this model type, we relied on the BIC and SSABIC (Sample Size Adjusted BIC) to select the two-class four known group model. Assessment of each knownclass individually likewise supported the choice of two classes. Figure 1 below presents the 1-class model, while Figure 2 presents the optimal 2-class model:

Figure 1: Latent class analysis of different MI types, 4 MI groups with 1 latent class per group.

Figure 2: Latent class analysis of different MI types, 4 MI groups with 2 latent classes per group.

MI = myocardial infarction, AcOcc = acute occlusion, NSTEMI = non-ST elevation myocardial infarction, STEMI = ST elevation myocardial infarction, OOH CA = out of hours cardiac arrest, NYHA = New York Heart Association heat failure classification, Trop +ve = troponin positive, LVEF mod/poor = left ventricular ejection fraction moderate/poor, Prev. MI = previous myocardial infarction, Prev. CABG = previous coronary artery bypass graft, Hist. CAD = history of coronary artery disease, valvular HD = valvular heart disease, Prev. CVE = previous cerebrovascular event, Per. Vasc. Dis. = peripheral vascular disease, hyperchol. = hypercholesteraemia, Hist. renal = history of renal disease, ECG ischaemia = electrocardiogram ischaemia.

Analysing the 2-class model, the “Model Test” option was then used to perform a Wald chi-square test of parameter equalities for each indicator across the 8 classes:

Table 4: Wald chi-square tests of difference for indicators across classes

| **Indicator** | **Wald Test Value** | **Degrees of freedom** | ***P*** |
| --- | --- | --- | --- |
| 1. Gender | 10.159 | 7 | 0.1797 |
| 2. Cardiogenic Shock | 34.159 | 7 | 0.0000 |
| 3. Out of Hospital Cardiac Arrest | 20.861 | 7 | 0.0040 |
| 4. Angina | 122.086 | 7 | 0.0000 |
| 5. NYHA | 7.394 | 7 | 0.3890 |
| 6. Troponin +ve | 212.661 | 7 | 0.0000 |
| 7. LVEF moderate/poor | 122.304 | 7 | 0.0000 |
| 8. Previous MI | 52.552 | 7 | 0.0000 |
| 9. Previous CABG | 332.186 | 7 | 0.0000 |
| 10. Diabetes | 62.047 | 7 | 0.0000 |
| 11. History of CAD | 40.808 | 7 | 0.0000 |
| 12. Valvular HD | 340.693 | 7 | 0.0000 |
| 13. Previous CVE | 36.721 | 7 | 0.0000 |
| 14. Peripheral Vascular Disease | 292.638 | 7 | 0.0000 |
| 15. Hypercholesterolemia | 171.670 | 7 | 0.0000 |
| 16. Hypertension | 69.452 | 7 | 0.0000 |
| 17. History of renal disease | 306.280 | 7 | 0.0000 |
| 18. ECG ischaemia | 3.824 | 7 | 0.7998 |
| 19. Age | 153.115 | 7 | 0.0000 |

To determine between which classes and for which indicators the significant differences arose, the “Model Constraint” option was used to perform z-tests on the indicator thresholds. Use of the “cinterval” option generated robust confidence intervals, from which standard errors and hence z scores and p-values can be derived. The Holm-Bonferroni correction was applied by ranking tests by increasing p-value, and testing whether they exceed $P_{k}<\frac{\alpha}{m+1-k}$ where m is the number of tests performed^3^.

Table 4 below presents the significant results found. MI groups (knownclass classes) are coded according to N=no acute occlusion/Y=acute occlusion, N=NSTEMI/S=STEMI; A and B denote different classes in a group; and indicators are numbered as in Table 3 above. Thus for example NNBYSA6 denotes class 2 of the non-occluded NSTEMI group compared against class 1 of the acutely occluded STEMI group. Figure 3 below uses the data presented in table 4 to select those indicators differing significantly between the acutely occluded STEMI and non-acutely occluded STEMI groups, extracting indicator probabilities from the model to illustrate that similarly to NSTEMI no indicators usefully distinguish acute occlusion from its absence.

Figure 3: Indicators differing significantly for occluded and non-occluded STEMI classes

Table 5: z tests of difference for Indicator/class pairs, Holm-Bonferroni correction applied

|  | **Confidence intervals** | | | | | | |  |  |  |  |  |  |
| --- | --- | --- | --- | --- | --- | --- | --- | --- | --- | --- | --- | --- | --- |
| **Indicator and classes compared** | **Lower 0.50%** | **Lower 2.50%** | **Lower 5%** | **Estimate** | **Upper 5%** | **Upper 2.50%** | **Upper 0.50%** | **Standard Error** | **abs z** | **Derived *p*** | **Rank** | **Holm-Bonferroni rejection criteria** |  |
| NNBYSA6 | 11.057 | 11.679 | 11.997 | 13.658 | 15.32 | 15.638 | 16.26 | 1.009949 | 13.52346 | 5.59703E-38 | 1 | 9.3985E-05 | sig |
| NNAYSA6 | 10.909 | 11.536 | 11.856 | 13.528 | 15.201 | 15.521 | 16.147 | 1.016582 | 13.30734 | 7.29196E-37 | 2 | 9.4162E-05 | sig |
| NNANSB9 | -15.977 | -15.348 | -15.026 | -13.344 | -11.663 | -11.341 | -10.711 | 1.022194 | 13.05428 | 1.40256E-35 | 3 | 9.434E-05 | sig |
| NNAYSB9 | -15.977 | -15.348 | -15.026 | -13.344 | -11.663 | -11.341 | -10.711 | 1.022194 | 13.05428 | 1.40256E-35 | 4 | 9.4518E-05 | sig |
| NNANNB17 | 10.298 | 10.932 | 11.256 | 12.948 | 14.639 | 14.963 | 15.597 | 1.028316 | 12.59146 | 2.72539E-33 | 5 | 9.4697E-05 | sig |
| NNBYSA17 | -15.597 | -14.963 | -14.639 | -12.948 | -11.256 | -10.932 | -10.298 | 1.028316 | 12.59146 | 2.72539E-33 | 6 | 9.4877E-05 | sig |
| NNANNB14 | -15.631 | -14.996 | -14.672 | -12.976 | -11.281 | -10.956 | -10.321 | 1.030612 | 12.59057 | 2.75242E-33 | 7 | 9.5057E-05 | sig |
| NNANSB14 | -15.631 | -14.996 | -14.672 | -12.976 | -11.281 | -10.956 | -10.321 | 1.030612 | 12.59057 | 2.75242E-33 | 8 | 9.5238E-05 | sig |
| NSANSB9 | -15.796 | -15.15 | -14.82 | -13.094 | -11.368 | -11.038 | -10.392 | 1.04898 | 12.48261 | 9.17094E-33 | 9 | 9.542E-05 | sig |
| NSAYSB9 | -15.796 | -15.15 | -14.82 | -13.094 | -11.368 | -11.038 | -10.392 | 1.04898 | 12.48261 | 9.17094E-33 | 10 | 9.5602E-05 | sig |
| NNBYNA15 | -15.299 | -14.672 | -14.351 | -12.676 | -11 | -10.68 | -10.052 | 1.018367 | 12.44737 | 1.35541E-32 | 11 | 9.5785E-05 | sig |
| NNAYNA15 | -15.394 | -14.762 | -14.439 | -12.751 | -11.063 | -10.74 | -10.108 | 1.02602 | 12.42763 | 1.68641E-32 | 12 | 9.5969E-05 | sig |
| NNANSB17 | 10.177 | 10.822 | 11.152 | 12.875 | 14.599 | 14.929 | 15.574 | 1.047704 | 12.28878 | 7.76672E-32 | 13 | 9.6154E-05 | sig |
| NSBYSA17 | -15.574 | -14.929 | -14.599 | -12.875 | -11.152 | -10.822 | -10.177 | 1.047704 | 12.28878 | 7.76672E-32 | 14 | 9.6339E-05 | sig |
| YNAYNB15 | 10.314 | 10.985 | 11.328 | 13.121 | 14.914 | 15.257 | 15.929 | 1.089796 | 12.03987 | 1.15287E-30 | 15 | 9.6525E-05 | sig |
| NSAYSA6 | 9.967 | 10.62 | 10.954 | 12.697 | 14.441 | 14.775 | 15.428 | 1.059949 | 11.97888 | 2.2153E-30 | 16 | 9.6712E-05 | sig |
| NNBNSA14 | 9.9 | 10.555 | 10.89 | 12.64 | 14.39 | 14.725 | 15.38 | 1.063776 | 11.88221 | 6.1984E-30 | 17 | 9.6899E-05 | sig |
| NSANSB14 | -15.38 | -14.725 | -14.39 | -12.64 | -10.89 | -10.555 | -9.9 | 1.063776 | 11.88221 | 6.1984E-30 | 18 | 9.7087E-05 | sig |
| YNBYSA6 | 10.119 | 10.796 | 11.143 | 12.951 | 14.76 | 15.106 | 15.783 | 1.09949 | 11.7791 | 1.84139E-29 | 19 | 9.7276E-05 | sig |
| YSAYSB6 | -15.484 | -14.817 | -14.476 | -12.695 | -10.913 | -10.572 | -9.905 | 1.082908 | 11.72306 | 3.31538E-29 | 20 | 9.7466E-05 | sig |
| NSAYNA15 | -15.204 | -14.543 | -14.205 | -12.439 | -10.673 | -10.335 | -9.674 | 1.073469 | 11.58766 | 1.3581E-28 | 21 | 9.7656E-05 | sig |
| NSBYSA6 | 9.171 | 9.839 | 10.181 | 11.965 | 13.75 | 14.091 | 14.759 | 1.084694 | 11.03076 | 3.81992E-26 | 22 | 9.7847E-05 | sig |
| NNBYSA14 | 9.243 | 9.955 | 10.32 | 12.223 | 14.127 | 14.492 | 15.204 | 1.157398 | 10.56076 | 3.6461E-24 | 23 | 9.8039E-05 | sig |
| NSBYSA14 | 9.243 | 9.955 | 10.32 | 12.223 | 14.127 | 14.492 | 15.204 | 1.157398 | 10.56076 | 3.6461E-24 | 24 | 9.8232E-05 | sig |
| NSBYSA9 | 9.252 | 9.967 | 10.333 | 12.245 | 14.156 | 14.522 | 15.238 | 1.16199 | 10.53796 | 4.52729E-24 | 25 | 9.8425E-05 | sig |
| YSAYSB9 | -15.238 | -14.522 | -14.156 | -12.245 | -10.333 | -9.967 | -9.252 | 1.16199 | 10.53796 | 4.52729E-24 | 26 | 9.8619E-05 | sig |
| NSBYNB9 | 9.247 | 9.969 | 10.339 | 12.269 | 14.199 | 14.568 | 15.291 | 1.173214 | 10.4576 | 9.67599E-24 | 27 | 9.8814E-05 | sig |
| YNBYSB9 | -15.291 | -14.568 | -14.199 | -12.269 | -10.339 | -9.969 | -9.247 | 1.173214 | 10.4576 | 9.67599E-24 | 28 | 9.901E-05 | sig |
| NNANSB12 | -14.039 | -13.375 | -13.035 | -11.261 | -9.487 | -9.148 | -8.483 | 1.078316 | 10.44313 | 1.10869E-23 | 29 | 9.9206E-05 | sig |
| NNAYNA12 | -14.039 | -13.375 | -13.035 | -11.261 | -9.487 | -9.148 | -8.483 | 1.078316 | 10.44313 | 1.10869E-23 | 30 | 9.9404E-05 | sig |
| NNAYNB12 | -14.039 | -13.375 | -13.035 | -11.261 | -9.487 | -9.148 | -8.483 | 1.078316 | 10.44313 | 1.10869E-23 | 31 | 9.9602E-05 | sig |
| NNAYSB12 | -14.039 | -13.375 | -13.035 | -11.261 | -9.487 | -9.148 | -8.483 | 1.078316 | 10.44313 | 1.10869E-23 | 32 | 9.98E-05 | sig |
| YNAYNB4 | 9.521 | 10.267 | 10.649 | 12.643 | 14.638 | 15.019 | 15.766 | 1.212245 | 10.42941 | 1.26133E-23 | 33 | 0.0001 | sig |
| NNAYNB17 | 9.501 | 10.249 | 10.631 | 12.628 | 14.625 | 15.007 | 15.755 | 1.213776 | 10.4039 | 1.60246E-23 | 34 | 0.0001002 | sig |
| YNBYSA17 | -15.755 | -15.007 | -14.625 | -12.628 | -10.631 | -10.249 | -9.501 | 1.213776 | 10.4039 | 1.60246E-23 | 35 | 0.0001004 | sig |
| NSANSB12 | -14.402 | -13.703 | -13.346 | -11.48 | -9.615 | -9.258 | -8.559 | 1.133929 | 10.12409 | 2.13619E-22 | 36 | 0.0001006 | sig |
| NSAYNA12 | -14.402 | -13.703 | -13.346 | -11.48 | -9.615 | -9.258 | -8.559 | 1.133929 | 10.12409 | 2.13619E-22 | 37 | 0.00010081 | sig |
| NSAYNB12 | -14.402 | -13.703 | -13.346 | -11.48 | -9.615 | -9.258 | -8.559 | 1.133929 | 10.12409 | 2.13619E-22 | 38 | 0.00010101 | sig |
| NSAYSB12 | -14.402 | -13.703 | -13.346 | -11.48 | -9.615 | -9.258 | -8.559 | 1.133929 | 10.12409 | 2.13619E-22 | 39 | 0.00010121 | sig |
| NNAYSB17 | 8.902 | 9.629 | 10.001 | 11.941 | 13.882 | 14.253 | 14.98 | 1.179592 | 10.12299 | 2.1578E-22 | 40 | 0.00010142 | sig |
| YSAYSB17 | 8.902 | 9.629 | 10.001 | 11.941 | 13.882 | 14.253 | 14.98 | 1.179592 | 10.12299 | 2.1578E-22 | 41 | 0.00010163 | sig |
| NNBYNA14 | 9.401 | 10.169 | 10.563 | 12.616 | 14.67 | 15.064 | 15.832 | 1.248724 | 10.10311 | 2.58739E-22 | 42 | 0.00010183 | sig |
| NSBYNA14 | 9.401 | 10.169 | 10.563 | 12.616 | 14.67 | 15.064 | 15.832 | 1.248724 | 10.10311 | 2.58739E-22 | 43 | 0.00010204 | sig |
| NNBNSB9 | -14.036 | -13.33 | -12.969 | -11.084 | -9.199 | -8.838 | -8.133 | 1.145918 | 9.672591 | 1.21641E-20 | 44 | 0.00010225 | sig |
| NNBYSB9 | -14.036 | -13.33 | -12.969 | -11.084 | -9.199 | -8.838 | -8.133 | 1.145918 | 9.672591 | 1.21641E-20 | 45 | 0.00010246 | sig |
| NNAYNB4 | 8.028 | 8.731 | 9.091 | 10.969 | 12.848 | 13.207 | 13.911 | 1.141837 | 9.606452 | 2.16791E-20 | 46 | 0.00010267 | sig |
| YNAYSA6 | 8.871 | 9.675 | 10.086 | 12.233 | 14.38 | 14.792 | 15.595 | 1.305357 | 9.371382 | 1.64141E-19 | 47 | 0.00010288 | sig |
| NSBYNA9 | 8.927 | 9.74 | 10.157 | 12.33 | 14.503 | 14.92 | 15.733 | 1.321429 | 9.330811 | 2.31704E-19 | 48 | 0.00010309 | sig |
| YNAYSB9 | -15.733 | -14.92 | -14.503 | -12.33 | -10.157 | -9.74 | -8.927 | 1.321429 | 9.330811 | 2.31704E-19 | 49 | 0.00010331 | sig |
| NSANSB19 | -17.211 | -16.32 | -15.863 | -13.481 | -11.099 | -10.643 | -9.751 | 1.448214 | 9.308705 | 2.7942E-19 | 50 | 0.00010352 | sig |
| NSBYSA19 | 9.586 | 10.551 | 11.044 | 13.622 | 16.201 | 16.694 | 17.659 | 1.567092 | 8.692535 | 4.38581E-17 | 51 | 0.00010373 | sig |
| NNBNSA19 | 9.334 | 10.294 | 10.785 | 13.348 | 15.912 | 16.403 | 17.362 | 1.558418 | 8.565094 | 1.19971E-16 | 52 | 0.00010395 | sig |
| NNBYNB4 | 7.117 | 7.869 | 8.253 | 10.262 | 12.27 | 12.655 | 13.407 | 1.220918 | 8.405148 | 4.16168E-16 | 53 | 0.00010417 | sig |
| NSBYNA15 | -14.217 | -13.402 | -12.984 | -10.805 | -8.626 | -8.209 | -7.393 | 1.324745 | 8.156287 | 2.76298E-15 | 54 | 0.00010438 | sig |
| YNAYSB15 | 7.573 | 8.41 | 8.838 | 11.074 | 13.31 | 13.738 | 14.575 | 1.359184 | 8.147538 | 2.95036E-15 | 55 | 0.0001046 | sig |
| NNBYSA19 | 9.204 | 10.229 | 10.753 | 13.489 | 16.226 | 16.75 | 17.774 | 1.66352 | 8.108707 | 3.94466E-15 | 56 | 0.00010482 | sig |
| NSBYNB4 | 7.061 | 7.848 | 8.251 | 10.353 | 12.454 | 12.857 | 13.644 | 1.277806 | 8.102168 | 4.14188E-15 | 57 | 0.00010504 | sig |
| NSAYSB19 | -21.153 | -19.906 | -19.268 | -15.938 | -12.608 | -11.97 | -10.723 | 2.02449 | 7.872601 | 2.24511E-14 | 58 | 0.00010526 | sig |
| YSAYSB19 | -21.361 | -20.098 | -19.452 | -16.079 | -12.706 | -12.06 | -10.797 | 2.05051 | 7.841463 | 2.81406E-14 | 59 | 0.00010549 | sig |
| NNANSB19 | -14.161 | -13.311 | -12.876 | -10.605 | -8.334 | -7.899 | -7.049 | 1.380612 | 7.681375 | 8.8745E-14 | 60 | 0.00010571 | sig |
| NNBNSA16 | -3.084 | -2.897 | -2.801 | -2.301 | -1.8 | -1.705 | -1.517 | 0.304082 | 7.567047 | 1.98931E-13 | 61 | 0.00010593 | sig |
| NNBYNB14 | 7.192 | 8.08 | 8.534 | 10.907 | 13.28 | 13.734 | 14.623 | 1.442347 | 7.561981 | 2.06124E-13 | 62 | 0.00010616 | sig |
| NSBYNB14 | 7.192 | 8.08 | 8.534 | 10.907 | 13.28 | 13.734 | 14.623 | 1.442347 | 7.561981 | 2.06124E-13 | 63 | 0.00010638 | sig |
| NNANSA17 | 6.862 | 7.722 | 8.162 | 10.46 | 12.758 | 13.199 | 14.059 | 1.397194 | 7.486434 | 3.49176E-13 | 64 | 0.00010661 | sig |
| NSAYSA17 | -14.059 | -13.199 | -12.758 | -10.46 | -8.162 | -7.722 | -6.862 | 1.397194 | 7.486434 | 3.49176E-13 | 65 | 0.00010684 | sig |
| NNAYNA17 | 7.067 | 7.977 | 8.443 | 10.874 | 13.305 | 13.771 | 14.681 | 1.478061 | 7.356935 | 8.52409E-13 | 66 | 0.00010707 | sig |
| YNAYSA17 | -14.681 | -13.771 | -13.305 | -10.874 | -8.443 | -7.977 | -7.067 | 1.478061 | 7.356935 | 8.52409E-13 | 67 | 0.0001073 | sig |
| NSBYSA12 | 7.001 | 7.912 | 8.378 | 10.812 | 13.246 | 13.712 | 14.623 | 1.479592 | 7.307421 | 1.19467E-12 | 68 | 0.00010753 | sig |
| YNAYSA12 | 7.001 | 7.912 | 8.378 | 10.812 | 13.246 | 13.712 | 14.623 | 1.479592 | 7.307421 | 1.19467E-12 | 69 | 0.00010776 | sig |
| YNBYSA12 | 7.001 | 7.912 | 8.378 | 10.812 | 13.246 | 13.712 | 14.623 | 1.479592 | 7.307421 | 1.19467E-12 | 70 | 0.00010799 | sig |
| YSAYSB12 | -14.623 | -13.712 | -13.246 | -10.812 | -8.378 | -7.912 | -7.001 | 1.479592 | 7.307421 | 1.19467E-12 | 71 | 0.00010823 | sig |
| NSBYNA19 | 9.294 | 10.557 | 11.203 | 14.578 | 17.952 | 18.598 | 19.861 | 2.051276 | 7.106798 | 4.59382E-12 | 72 | 0.00010846 | sig |
| YNAYNB19 | -19.995 | -18.687 | -18.018 | -14.525 | -11.031 | -10.362 | -9.055 | 2.123724 | 6.839399 | 2.6253E-11 | 73 | 0.0001087 | sig |
| YNAYSB19 | -23.451 | -21.917 | -21.132 | -17.034 | -12.936 | -12.151 | -10.617 | 2.491327 | 6.837321 | 2.66048E-11 | 74 | 0.00010893 | sig |
| NNANNB10 | -3.074 | -2.872 | -2.769 | -2.229 | -1.69 | -1.586 | -1.384 | 0.328061 | 6.794463 | 3.4983E-11 | 75 | 0.00010917 | sig |
| NNBYSB14 | 6.5 | 7.451 | 7.938 | 10.481 | 13.023 | 13.51 | 14.461 | 1.545663 | 6.780908 | 3.81351E-11 | 76 | 0.00010941 | sig |
| NSBYSB14 | 6.5 | 7.451 | 7.938 | 10.481 | 13.023 | 13.51 | 14.461 | 1.545663 | 6.780908 | 3.81351E-11 | 77 | 0.00010965 | sig |
| NNBYNA19 | 8.901 | 10.226 | 10.904 | 14.444 | 17.985 | 18.663 | 19.988 | 2.152296 | 6.710973 | 5.93703E-11 | 78 | 0.00010989 | sig |
| NSAYNB19 | -18.62 | -17.379 | -16.744 | -13.428 | -10.113 | -9.478 | -8.237 | 2.015561 | 6.662164 | 8.06666E-11 | 79 | 0.00011013 | sig |
| NNBNSA7 | 1.08 | 1.243 | 1.327 | 1.763 | 2.199 | 2.283 | 2.446 | 0.265306 | 6.645154 | 8.97196E-11 | 80 | 0.00011038 | sig |
| NSAYNB4 | 6.205 | 7.144 | 7.625 | 10.134 | 12.644 | 13.125 | 14.064 | 1.525765 | 6.641913 | 9.15539E-11 | 81 | 0.00011062 | sig |
| NNAYSB19 | -18.166 | -16.945 | -16.321 | -13.062 | -9.802 | -9.178 | -7.957 | 1.981378 | 6.592383 | 1.24602E-10 | 82 | 0.00011086 | sig |
| NNANNB16 | 1.156 | 1.337 | 1.43 | 1.916 | 2.401 | 2.494 | 2.676 | 0.295153 | 6.491547 | 2.31891E-10 | 83 | 0.00011111 | sig |
| NNANNB19 | -14.641 | -13.644 | -13.135 | -10.472 | -7.809 | -7.299 | -6.302 | 1.618622 | 6.469699 | 2.65001E-10 | 84 | 0.00011136 | sig |
| YNBYSA19 | 8.058 | 9.375 | 10.05 | 13.57 | 17.09 | 17.764 | 19.082 | 2.140051 | 6.34097 | 5.77124E-10 | 85 | 0.00011161 | sig |
| YNAYSA15 | 6.388 | 7.46 | 8.009 | 10.875 | 13.741 | 14.29 | 15.362 | 1.742347 | 6.241581 | 1.04269E-09 | 86 | 0.00011186 | sig |
| NNBNSB12 | -12.638 | -11.745 | -11.288 | -8.903 | -6.518 | -6.062 | -5.169 | 1.449745 | 6.14108 | 1.88054E-09 | 87 | 0.00011211 | sig |
| NNBYNA12 | -12.638 | -11.745 | -11.288 | -8.903 | -6.518 | -6.062 | -5.169 | 1.449745 | 6.14108 | 1.88054E-09 | 88 | 0.00011236 | sig |
| NNBYNB12 | -12.638 | -11.745 | -11.288 | -8.903 | -6.518 | -6.062 | -5.169 | 1.449745 | 6.14108 | 1.88054E-09 | 89 | 0.00011261 | sig |
| NNBYSB12 | -12.638 | -11.745 | -11.288 | -8.903 | -6.518 | -6.062 | -5.169 | 1.449745 | 6.14108 | 1.88054E-09 | 90 | 0.00011287 | sig |
| NNANSB8 | -3.152 | -2.924 | -2.806 | -2.195 | -1.583 | -1.466 | -1.237 | 0.371939 | 5.901509 | 7.41508E-09 | 91 | 0.00011312 | sig |
| NNBNSB7 | 0.694 | 0.826 | 0.894 | 1.248 | 1.601 | 1.669 | 1.802 | 0.215051 | 5.803274 | 1.28363E-08 | 92 | 0.00011338 | sig |
| NNANNB8 | -2.313 | -2.134 | -2.043 | -1.565 | -1.088 | -0.996 | -0.818 | 0.290306 | 5.390861 | 1.1774E-07 | 93 | 0.00011364 | sig |
| NNAYNB19 | -15.595 | -14.389 | -13.773 | -10.552 | -7.332 | -6.715 | -5.509 | 1.957653 | 5.390128 | 1.18191E-07 | 94 | 0.0001139 | sig |
| NNBYSA16 | -3.076 | -2.836 | -2.713 | -2.07 | -1.428 | -1.305 | -1.064 | 0.390561 | 5.300065 | 1.88179E-07 | 95 | 0.00011416 | sig |
| NNBYNA16 | -3.259 | -3.002 | -2.87 | -2.182 | -1.493 | -1.361 | -1.104 | 0.418622 | 5.212334 | 2.94111E-07 | 96 | 0.00011442 | sig |
| NSANSB16 | 0.867 | 1.093 | 1.208 | 1.81 | 2.411 | 2.526 | 2.752 | 0.365561 | 4.951291 | 1.06938E-06 | 97 | 0.00011468 | sig |
| NNANSB16 | 0.676 | 0.855 | 0.947 | 1.425 | 1.902 | 1.994 | 2.173 | 0.290561 | 4.904302 | 1.34101E-06 | 98 | 0.00011494 | sig |
| NNANSB10 | -2.453 | -2.25 | -2.146 | -1.605 | -1.063 | -0.959 | -0.756 | 0.329337 | 4.873431 | 1.55445E-06 | 99 | 0.00011521 | sig |
| NNBNSA8 | 0.643 | 0.833 | 0.93 | 1.437 | 1.943 | 2.04 | 2.23 | 0.307908 | 4.666976 | 4.08995E-06 | 100 | 0.00011547 | sig |
| NNBYSB7 | 0.765 | 0.992 | 1.109 | 1.716 | 2.323 | 2.44 | 2.667 | 0.369388 | 4.645525 | 4.51324E-06 | 101 | 0.00011574 | sig |
| NNBNSA11 | -2.156 | -1.968 | -1.872 | -1.37 | -0.868 | -0.772 | -0.584 | 0.305102 | 4.490301 | 9.09967E-06 | 102 | 0.00011601 | sig |
| NSANSB8 | -3.269 | -2.981 | -2.834 | -2.066 | -1.298 | -1.151 | -0.864 | 0.466837 | 4.42553 | 1.21207E-05 | 103 | 0.00011628 | sig |
| NNBYSA7 | 1.008 | 1.355 | 1.533 | 2.462 | 3.391 | 3.569 | 3.917 | 0.564796 | 4.359097 | 1.6205E-05 | 104 | 0.00011655 | sig |
| NNANNB11 | 0.522 | 0.703 | 0.796 | 1.279 | 1.762 | 1.855 | 2.035 | 0.293878 | 4.352153 | 1.67009E-05 | 105 | 0.00011682 | sig |
| NNBYNB7 | 0.577 | 0.783 | 0.889 | 1.441 | 1.993 | 2.099 | 2.305 | 0.335714 | 4.29234 | 2.16162E-05 | 106 | 0.0001171 | sig |
| NNANNB13 | -2.348 | -2.136 | -2.027 | -1.46 | -0.893 | -0.784 | -0.572 | 0.344898 | 4.233136 | 2.7823E-05 | 107 | 0.00011737 | sig |
| NSBYNA16 | -2.79 | -2.527 | -2.393 | -1.69 | -0.988 | -0.854 | -0.591 | 0.426786 | 3.959833 | 8.59114E-05 | 108 | 0.00011765 | sig |

References

1. Nylund, K.L., Asparouhov, T., & Muthén, B. (2007). Deciding on the number of classes in latent class analysis and growth mixture modeling. A Monte Carlo simulation study. Structural Equation Modeling, 14, 535-569.
2. Collins, L.M. & Lanza, S.T. (2009). Latent class and latent transition analysis: With applications in the social, behavioral, and health sciences, pp 121-125. Wiley Series in Probability and Statistics Ser., vol. 718. John Wiley & Sons.
3. Holm, S. (1979). "A simple sequentially rejective multiple test procedure". Scandinavian Journal of Statistics. 6 (2): 65–70.
